# Detection and Monitoring of *Mycobacterium leprae* Infection in Nine Banded Armadillos (*Dasypus novemcinctus*) Using a Quantitative Rapid Test

**DOI:** 10.3389/fmicb.2021.763289

**Published:** 2021-10-28

**Authors:** Zijie Zhou, Maria Pena, Anouk van Hooij, Louise Pierneef, Danielle de Jong, Roena Stevenson, Rachel Walley, Paul L. A. M. Corstjens, Richard Truman, Linda Adams, Annemieke Geluk

**Affiliations:** ^1^Department of Infectious Diseases, Leiden University Medical Center, Leiden, Netherlands; ^2^U.S. Department of Health and Human Services, Health Resources and Services Administration, Health Systems Bureau, National Hansen Disease Programme (NHDP), Baton Rouge, LA, United States; ^3^Department Cell and Chemical Biology, Leiden University Medical Center, Leiden, Netherlands; ^4^Department of Pathobiological Sciences, Louisiana State University, Baton Rouge, LA, United States

**Keywords:** antibodies, armadillo, diagnosis, lateral flow assay, leprosy, *M. leprae*, PGL-I, POC

## Abstract

Leprosy is an infectious disease caused by *Mycobacterium leprae* with tropism for skin and peripheral nerves. Incessant transmission in endemic areas is still impeding elimination of leprosy. Although detection of *M. leprae* infection remains a challenge in asymptomatic individuals, the presence of antibodies specific for phenolglycolipid-I (PGL-I) correlate with bacterial load. Therefore, serosurveillance utilizing field-friendly tests detecting anti-PGL-I antibodies, can be applied to identify those who may transmit bacteria and to study (reduction of) *M. leprae* transmission. However, serology based on antibody detection cannot discriminate between past and present *M. leprae* infection in humans, nor can it detect individuals carrying low bacillary loads. In humans, anti-PGL-I IgM levels are long-lasting and usually detected in more individuals than anti-PGL-I IgG levels. Inherent to the characteristically long incubation time of leprosy, IgM/IgG relations (antibody kinetics) in leprosy patients and infected individuals are not completely clear. To investigate the antibody response directly after infection, we have measured antibody levels by ELISA, in longitudinal samples of experimentally *M. leprae* infected, susceptible nine-banded armadillos (*Dasypus novemcinctus*). In addition, we assessed the user- and field-friendly, low-cost lateral flow assay (LFA) utilizing upconverting reporter particles (UCP), developed for quantitative detection of human anti-PGL-I IgM (UCP-LFA), to detect treatment- or vaccination-induced changes in viable bacterial load. Our results show that serum levels of anti-PGL-I IgM, and to a lesser extent IgG, significantly increase soon after experimental *M. leprae* infection in armadillos. In view of leprosy phenotypes in armadillos, this animal model can provide useful insight into antibody kinetics in early infection in the various spectral forms of human leprosy. The UCP-LFA for quantitative detection of anti-PGL-I IgM allows monitoring the efficacy of vaccination and rifampin-treatment in the armadillo leprosy model, thereby providing a convenient tool to evaluate the effects of drugs and vaccines and new diagnostics.

## Introduction

Leprosy is a severe, communicable disease characterized by skin lesions and peripheral nerve alterations associated with a wide-ranging spectrum of clinical symptoms. Worldwide, millions of people are coping with the long term consequences of this dermato-neurological disease and more than 200,000 new patients are annually diagnosed, making it the second most common mycobacterial disease after tuberculosis (WHO, 2020). The causative pathogens of leprosy, *Mycobacterium leprae* and *Mycobacterium lepromatosis*, are obligate intracellular bacteria which grow optimally in humans at lower body temperature and have a tropism for Schwann cells (Scollard et al., 2006; Eichelmann et al., 2013). If left untreated, leprosy can lead to irreversible nerve damage and lifelong handicaps and deformities in humans.

Leprosy elimination is still hampered by intense transmission of *Mycobacterium leprae*, which is not restricted to patients’ households (Ortuno-Gutierrez et al., 2019; Tió-Coma et al., 2020). At the individual level, detection of *M. leprae* infection in persons without symptoms is essential to detect patients at early, still well-treatable stages of leprosy. On a community scale, on the other hand, detection of *M. leprae* infection bears relevance to monitoring transmission in an area on the way to elimination of this disease, and can help to assess the effects of prophylactic community interventions. Interruption of *M. leprae* transmission is crucial and concomitantly are tests to detect asymptomatically infected individuals who can still perpetuate transmission.

Characteristic for leprosy is its disease spectrum, with on one end tuberculoid leprosy (TT) associated with Th1 and Th17 T cell immune responses, and on the other end lepromatous leprosy (LL) (Hungria et al., 2018; van Hooij and Geluk, 2021), the more severe type of leprosy with disseminated infection generally linked to anti-inflammatory Th2 responses (Salgame et al., 1991; Quaresma et al., 2015; Aarão et al., 2016). Moreover, LL patients, also referred to as multibacillary (MB) leprosy, display well detectable antibody levels, in particular IgM directed against *M. leprae* PGL-I, whereas IgG and IgA are detected mostly in lower levels. Although anti-PGL-I IgM seropositivity increases the risk of leprosy (Gormus et al., 2000), it does not predict disease (Richardus et al., 2017; TiemiNagao-Dias et al., 2019). The much less frequently observed anti-PGL-I IgG seropositivity, however, has been described to be associated with disease development (TiemiNagao-Dias et al., 2019). As severe infection is associated with high IgM antibody levels, it is improbable that these convey protective anti-*M. leprae* immunity. Given the correlation between anti-PGL-I IgM levels and bacterial load (Tió-Coma et al., 2020; van Hooij et al., 2020), this humoral immunity biomarker is useful to detect *M. leprae* infection as well as diagnose MB patients (van Hooij et al., 2017, 2019, 2021). Studies in humans on the kinetics of antibody production directly after *M. leprae* infection, including quantitative and qualitative differences between IgM and IgG, have been considerably constrained due to the facts that anti-PGL-I immunity cannot discriminate between past and present *M. leprae* infection (Pierneef et al., 2021), most humans do not develop disease after *M. leprae* infection and leprosy has a long incubation time (Scollard et al., 2006).

Six-banded (*Euphractus sexcinctus*) as well as nine-banded (*Dasypus novemcinctus*) armadillos represent a potential environmental reservoir for zoonotic transmission of *M. leprae* (Truman et al., 2011; Sharma et al., 2015; da Silva et al., 2018; da Silva Ferreira et al., 2020). In 1971, it was demonstrated that the nine-banded armadillo developed leprosy after inoculation with *M. leprae* (Job and Truman, 2000). Due to their low body temperature of 32–35°C, the infection disseminates to all body tissues resulting in high bacterial loads in the spleen, liver, and lymph nodes. Similar to humans, susceptibility to leprosy varies resulting in resistance in 15–20% of *M. leprae* infected armadillos (Balamayooran et al., 2015). Immunologically, leprosy in armadillos also resembles human leprosy as shown by the IFN-γ responses to a variety of recombinant *M. leprae* proteins in peripheral blood mononuclear cells (PBMCs) from *M. leprae* infected armadillos (Geluk et al., 2011; Pena et al., 2011). Thus, armadillos provide a useful animal model to study leprosy-specific neuropathy (Adams et al., 2012; Sharma et al., 2013; Truman et al., 2014; Balamayooran et al., 2015; Oliveira et al., 2019), skin test reagents (Duthie et al., 2020b) and vaccines against leprosy (Duthie et al., 2018).

ID93/GLA-SE is a tuberculosis candidate vaccine, composed of a fusion protein (ID93) consisting of four *Mycobacterium tuberculosis* antigens (Rv1813c, Rv2608, Rv3619c, and Rv3620c) combined with a synthetic Toll-like receptor 4 agonist: GLA-SE adjuvant (Glucopyranosyl Lipid Adjuvant in stable emulsion) (Bertholet et al., 2008; Day et al., 2021). In mice ID93/GLA-SE provides cross-protection against *M. leprae* infection (Duthie et al., 2014). LepVax (LEP-F1 adjuvanted with GLA-SE), a leprosy candidate vaccine (Duthie et al., 2020a) was formulated with a fusion protein LEP-F1 (named ML89 during preclinical development; comprised of ML2351, ML2055, ML2380, and ML2028 antigens) combined with GLA-SE. LepVax was tested in armadillos and showed reduction of *M. leprae* bacillary load and delayed *M. leprae*-induced motor nerve damage (Duthie et al., 2018).

To gain more insight into the kinetics of anti-PGL-I antibody formation directly after *M. leprae* infection and the meaning of differential seropositivity for IgM and IgG, we have measured both antibody levels by ELISA in longitudinal samples of experimentally *M. leprae* inoculated, susceptible nine-banded armadillos. In addition, we have evaluated the potential of a point-of-care (POC) test for quantitative detection of human anti-PGL-I IgM (UCP-LFA) for detection of anti-PGL-I antibodies in *M. leprae* infected armadillos early after infection, monitoring antibody levels during the course of infection and after treatment as well as to discriminate between resistant and susceptible animals. Successful use of the UCP-LFA in armadillos with known time points of infection, bears relevance to similar applications in humans especially in remote and resource limited leprosy endemic settings.

## Materials and Methods

### Ethics

The present studies were performed on armadillo serum samples, which were banked from previous *M. leprae* infection experiments at the National Hansen’s Disease Program (NHDP) (Duthie et al., 2018; Hazbón et al., 2018). Armadillo experiments were conducted in accordance with procedures approved by the NHDP Animal Care and Use Committee [Assurance No. D16-00019 (A3032-01)]. Based on estimates of anticipated variation of rodent models of *M. leprae* infection and on prior experience with the experimental systems, the armadillos were distributed randomly in different groups. The resulting group sizes provide proper statistical analysis while allowing minimal animal usage.

### Animal Husbandry

Wild-caught armadillos are mainly adult animals that were captured alive in the wild and brought to the NHDP vivarium where they were housed in modified rabbit cages (Pena et al., 2016) for conditioning before experimental inoculation. During conditioning, the animals were treated for bacterial and parasitic infections and microchipped. Before experimental inoculation, the animals were screened for serum antibodies to *M. leprae* PGL-I to determine whether there was natural infection with *M. leprae*. Each animal was also lepromin tested to determine immune responsiveness to *M. leprae.* The lepromin test coincides with histopathology which ranges across the entire spectrum of leprosy (Adams et al., 2012). Only armadillos showing a negative lepromin response (indicating susceptibility to *M. leprae*) were experimentally infected with *M. leprae*. Regarding captive-born armadillos, pregnant females were caught in the wild and kept in captivity where they give birth to four genetically identical siblings. These young armadillos were brought to the NHDP vivarium at about 4 months of age and placed in rabbit cages for a period of conditioning of about 12–24 months. At this time the animals were microchipped, dewormed, screened for anti-PGL-I, and lepromin tested. The average age at infection was about 24 months.

### Armadillo Cohort

**FIGURE 1:**
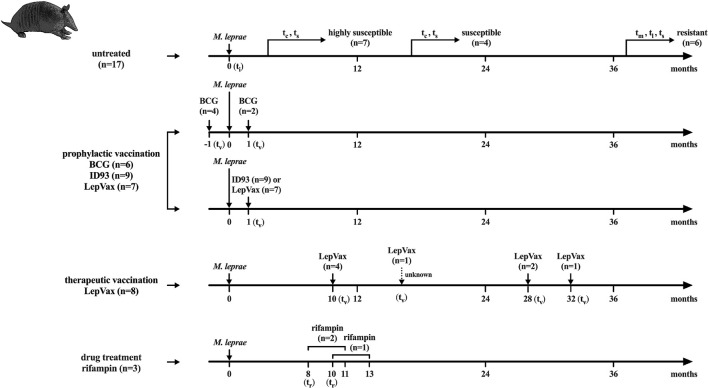
Flowchart of infection, treatment and sampling, Sample collected at several time points from 52 nine-banded armadillos (*Dasypus novemcinctus*) infected with 1 × 10^9^ *M. leprae* bacilli from strains: NHDP 63 (*n* = 33), NHDP 98 (*n* = 7), or Brazil 4923 (*n* = 11). One animal was infected in the wild. *M. leprae* infected armadillos that were untreated and unvaccinated (*n* = 17) were divided into 3 groups according to *M. leprae* dissemination and disease progression after inoculation: highly susceptible (within 12 months; *n* = 7), susceptible (within 24 months; *n* = 4), and resistant (after more than 36 months; *n* = 6); Treated animals were divided into: Armadillos vaccinated with BCG 1 month pre- (*n* = 6) or post- (*n* = 2) infection; Armadillos prophylactically vaccinated with ID93 (*n* = 9) or LepVax (*n* = 7) 1 month post-infection; Armadillos therapeutically vaccinated LepVax (*n* = 8) 10–32 months post-infection; Armadillos treated for 3 months with rifampin 8 or 10 months post-infection (*n* = 3). For the animal that was infected in the wild and LepVax vaccinated, the inoculation time (t_i_) and interval between t_i_ were unknown. t_i_, time of inoculation; t_v_, time of vaccination; t_r_, time of rifampin treatment; t_c_, time of seroconversion (anti-PGL-I IgM ELISA > 0.45 OD_450__–__background_; for the highly susceptible and susceptible group); t_m_, mid-stage disease; t_l_, late-stage disease; t_s_, time at sacrifice (see Supplementary Table 1).

The armadillo cohort from which serum samples were analyzed contained 52 nine-banded armadillos (*Dasypus novemcinctus*) and was comprised of both captive-born and wild-caught animals which were experimentally infected with one of three strains of *M. leprae* [NHDP 63 (n = 33), NHDP 98 (n = 7), or Brazil 4923 (n = 11)], and one animal was infected in the wild. Out of the *M. leprae* infected armadillos, 17 were untreated and unvaccinated and could be divided further into three groups based on full dissemination time as described (Pena et al., 2016): highly susceptible (within 12 months), susceptible (within 24 months), and resistant (lack of dissemination at 36 months); BCG vaccination was given to 8 armadillos 1 month pre- (n = 6) or post- (n = 2) infection; 24 armadillos were vaccinated with ID93 or LepVax 1–32 months post-infection; three armadillos were treated for 3 months with rifampin 8 or 10 months post-infection. ID93 = Rv1813c, Rv2608, Rv3619c, and Rv3620c adjuvanted with GLA-SE; LepVax = ML2351, ML2055, ML2380, and ML2028 adjuvanted with GLA-SE. Sampling time points were divided into t_i_: time of inoculation; t_c_: time of seroconversion (anti-PGL-I IgM ELISA > 0.45 OD_450__–__background_; for highly susceptible and susceptible animals); t_v_: time of vaccination; t_r_: time of rifampin treatment; t_m_: mid-stage disease; t_l_: late-stage disease; t_s_: time at sacrifice (Figure 1 and Supplementary Tables 1, 2).

### Experimental *M. leprae* Inoculation

Three strains of *M. leprae* are cultivated in nude mouse footpads at the NHDP and serially passaged to maintain a high percent viability. *M. leprae* were harvested from the footpads, stored at 4°C, and used with 24 h. For inoculation, the armadillos were anesthetized and the skin at the injection site was cleaned with 70% ethanol. *M. leprae* were resuspended in saline at 1 × 10^9^ bacilli per 1 ml and injected slowly into the saphenous vein (Truman and Krahenbuhl, 2001; Wallace et al., 2021).

### Disease Progression

After experimental inoculation with *M. leprae*, a serum sample was collected every three months to screen for anti-PGL-I antibodies. When a positive ELISA assay was detected [corresponding to an optical density at 450 nm (OD_450__–__background_) of 0.45], the animals were followed for disease progression and dissemination by serology, hematology (hematocrit) and blood chemistry (lactate dehydrogenase; LDH). On average the animals were anti-PGL-I seropositive (anti-PGL-I IgM ELISA: OD_450__–__background_ > 0.45) at 9 months after *M. leprae* inoculation. By 18–24 months post-inoculation most animals develop a severe infection. The armadillos were humanely sacrificed when generalized dissemination of bacilli was reached and the animals were seropositive, had a hematocrit lower than 20 and LDH levels were higher than 2,000.

### Anti-PGL-I Antibodies ELISA

Levels of anti-*M. leprae* PGL-I IgM or -IgG in armadillos’ sera were measured by ELISAs described previously (Duthie et al., 2011; van Hooij et al., 2017, 2019; van Dijk et al., 2021); briefly, 96-well PolySorp NUNC plates (Thermo Fisher Scientific) were coated with 50 μl of the antigens ND-O-HSA (4 μg/ml, BEI Resources, NR-19347) in 100 mM Na_2_CO_3_/NaHCO_3_ buffer (pH 9.6) at 4° overnight. Coated plates were blocked with 200 μl PBS/1% BSA/0.05% Tween 80 per well for 1 h, 50 μl of 1:35 diluted sample was added and incubated for 2 h at room temperature. Then, 50 μl of anti-human IgG-HRP (1:4,000, Sigma, A0170) or IgM-HRP (1:8,000, Sigma, A6907) in 0.05%Tween 20/PBS was incubated for 2 h. In between each step the wells were washed 3 times with PBS/0.05% Tween 20. 50 μl of 3,3′,5,5′-Tetramethylbenzidine (TMB, Thermo Fisher Scientific, Rochester, NY) was added and the color reaction was stopped using H_2_SO_4_ after 10 min. Absorbance was determined at a wavelength of 450 nm. The optical density at 450 nm (OD_450_) of the samples was corrected with the background OD (0.1% BSA in coating buffer).

### Up-Converting Reporter Particles Conjugate

LFAs were performed with luminescent up-converting reporter particles (UCP) as a sensitive background-free label for quantitative detection of targeted analytes (Corstjens et al., 2003, 2005; Zuiderwijk et al., 2003). UCP nanomaterials (200 nm NaYF_4_:Yb^3+^, Er^3+^ particles, functionalized with carboxyl groups) were obtained from Intelligent Material Solutions Inc. (Princeton, New Jersey, United States). UCP conjugates were prepared with goat anti-human IgM (I0759, Sigma-Aldrich, St. Louis, Missouri, United States) at a concentration of 50 μg antibody per mg UCP according to the method described previously (Bobosha et al., 2014; Corstjens et al., 2019).

### Lateral Flow Strips

LF strips (4 mm width) for detection of anti-PGL-I IgM were produced as described previously (Corstjens et al., 2013; van Hooij et al., 2016, 2017, 2018, 2019). The test (T) line comprised 100 ng synthetic PGL-I (ND-O-HSA) and the flow control (FC) line comprised 100 ng rabbit anti-goat IgG (G4018, Sigma-Aldrich). UCP reporter conjugate (400 ng) was dried into the sample/conjugate-release pad using a buffer containing 5% (w/v) sucrose. LF strips were stored at ambient temperature in plastic containers with silica dry pad.

### Anti-PGL-I IgM UCP-LFA

Serum samples were analyzed using 50 μl of a 1:50 (anti-PGL-I IgM) dilution in assay buffer (100 mM Tris pH 8, 270 mM NaCl, 1% (w/v) BSA, 1% (v/v) Triton X-100). Diluted serum samples (50 μl) were applied to LF strips. Upon completion of LF, strips were analyzed with a UCP dedicated benchtop reader (UPCON; Labrox, Finland). Results are displayed as the ratio value (R) of T and FC signals (peak area) based on relative fluorescence units (RFUs).

### Statistical Analysis

Graphpad Prism version 9.0 for Windows (GraphPad Software, San Diego CA, United States) was used to perform Mann-Whitney *U*-tests, Wilcoxon matched-pairs tests, Kruskal-Wallis with Dunn’s correction for multiple testing, plot receiver operating characteristic (ROC) curves, calculate the area under the curve (AUC), and compute Spearman correlation coefficients. The optimal sensitivity and specificity were determined using Youden’s index (Fluss et al., 2005). The statistical significance level used was *p* < 0.05.

## Results

### Longitudinal Anti-PGL-I Antibody Levels in *M. leprae* Infected Armadillos

In this study, serum samples were analyzed that were previously collected in different experiments from *M. leprae* infected armadillo including. untreated, vaccinated, and drug-treated armadillos (Figure 1). To assess the kinetics of humoral anti-*M. leprae* responses in armadillos, we first assessed the levels of anti-PGL-I antibodies by ELISA in serum obtained from 17 armadillos that were experimentally infected with *M. leprae* [1 × 10^9^ bacilli; NHDP 63 (*n* = 9), NHDP 98 (*n* = 4), or Brazil 4923 (*n* = 4)]. Armadillos were sampled at multiple time points and divided into three categories according to the time of dissemination of *M. leprae* infection (Pena et al., 2016; Supplementary Table 2). The (highly) susceptible group is the highly susceptible- combined with the susceptible group. At the time of sacrifice (t_s_), bacillary burden in the liver and spleen of (highly) susceptible armadillos was higher than in resistant animals. Highly susceptible armadillos also had slightly increased numbers of bacteria compared to susceptible animals (Supplementary Figure 1).

**FIGURE 2:**
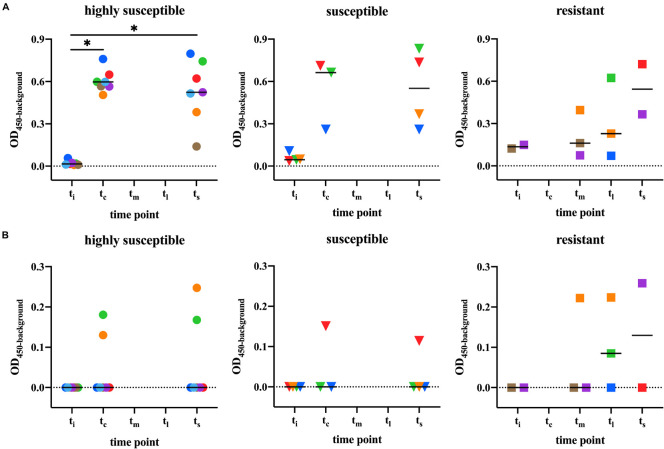
Anti-PGL-I antibody levels in M. leprae infected armadillos. Anti-PGL-I antibody levels were measured by ELISA in serum from *M. leprae* [1 × 10^9^ bacilli; NHDP 63 (*n* = 9), NHDP 98 (*n* = 4), or Brazil 4923 (*n* = 4)] infected armadillos. Sample derived from each animal is represented by the same symbol. After infection, seven armadillos disseminated before 12 months (highly susceptible; dots), four disseminated and progressed to disease within 24 months (susceptible; triangles), and six armadillos did not disseminate even 36 months post-infection (resistant; squares). ELISA results of anti-PGL-I IgM **(A)** and anti-PGL-I IgG **(B)** are displayed as optical density at 450 nm corrected for background OD values (OD_450__–__background_; *y-axis*). The median values per group are indicated by horizontal lines. Differences between pre-infection (t_i_) vs. post-infection (t_c_/t_m_/t_l_/t_s_) were determined by Wilcoxon matched-pairs test. *P*-values: ^∗^*p* < 0.05. t_i_, time of inoculation; t_c_, time of seroconversion (for highly susceptible and susceptible animals); t_m_, mid-stage disease; t_l_, late-stage disease; t_s_, time at sacrifice.

Before *M. leprae* infection (t_i_), no anti-PGL-I IgM or -IgG antibodies were detected in any of the armadillos (Figure 2). Upon infection with *M. leprae*, the anti-PGL-I IgM levels significantly increased in highly susceptible armadillos compared to pre-infection levels (t_i_ vs. t_c_, *p* < 0.05; t_i_ vs. t_s_, *p* < 0.05). In line with the classification of the armadillos, all susceptible animals became seropositive, but the antibody levels did not reach similarly high values (t_i_ vs. t_c_, *p* = 0.25; t_i_ vs. t_s_, *p* = 0.125). In resistant armadillos, on the other hand, after *M. leprae* infection, the animals remained seronegative during mid-stage disease (t_m_) while only one out of three became seropositive. At the late stage (t_l_) only two animals were tested which were seropositive (Figure 2A). In all animals, IgG levels were lower than IgM levels at all-time points. IgG after *M. leprae* infection was detected in six armadillos, three of which belonged to the resistant group. In the resistant group 50% (*n* = 3) showed detectable IgG, compared to 25% (*n* = 1) of the susceptible animals and 29% of the highly susceptible animals (Figure 2B).

The kinetics of anti-PGL-I IgM and IgG after *M. leprae* infection varied with susceptibility to *M. leprae* infection, as IgM levels were lower in the resistant- compared with highly susceptible and susceptible animals, while IgG levels were more frequently detected in resistant animals.

To analyze the correlation between anti-PGL-I antibodies and other blood parameters that indicate disease severity, we compared anti-PGL-I antibodies with white blood cell counts (WBC), hematocrit (HCT), lactate dehydrogenase (LDH), aspartate aminotransferase (AST), and alanine aminotransferase (ALT) from the same animals. However, there were no or only weak correlations between anti-PGL-I antibodies with the different blood values (*R*^2^ ≤ 0.17; Supplementary Figure 2), indicating that anti-PGL-I antibody levels provide improved diagnostic value for *M. leprae* infection.

### Anti-PGL-I IgM UCP-LFA Correlates With ELISA

**FIGURE 3:**
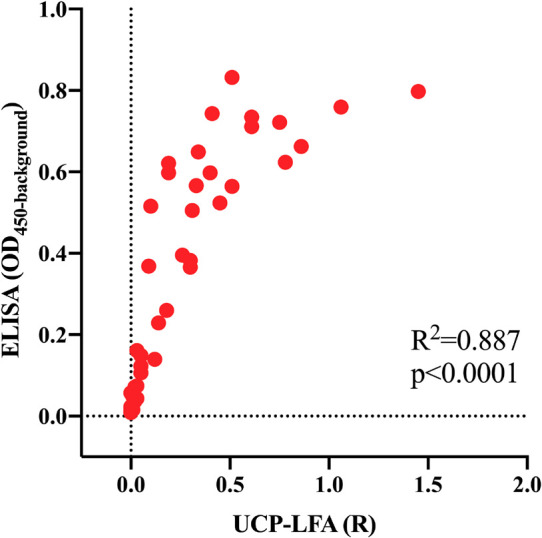
Correlation of anti-PGL-I IgM levels in *M. leprae* infected armadillos in UCP-LFA and ELISA. Serum samples (*n* = 41) of *M. leprae* [1 × 10^9^ bacilli; NHDP 63 (*n* = 9), NHDP 98 (*n* = 4), or Brazil 4923 (*n* = 4)] infected armadillos at multiple time points, were assessed for anti-PGL-I IgM by UCP-LFA, and for anti-PGL-I IgM by ELISA. UCP-LFA results are displayed as the Ratio value (R) between Test (T) and Flow-Control (FC) signal (*x*-axis); ELISA results are displayed as optical density at 450 nm corrected by background (OD_450__–__background_; *y*-axis). R^2^ is the square of the Spearman correlation coefficient.

**FIGURE 4:**
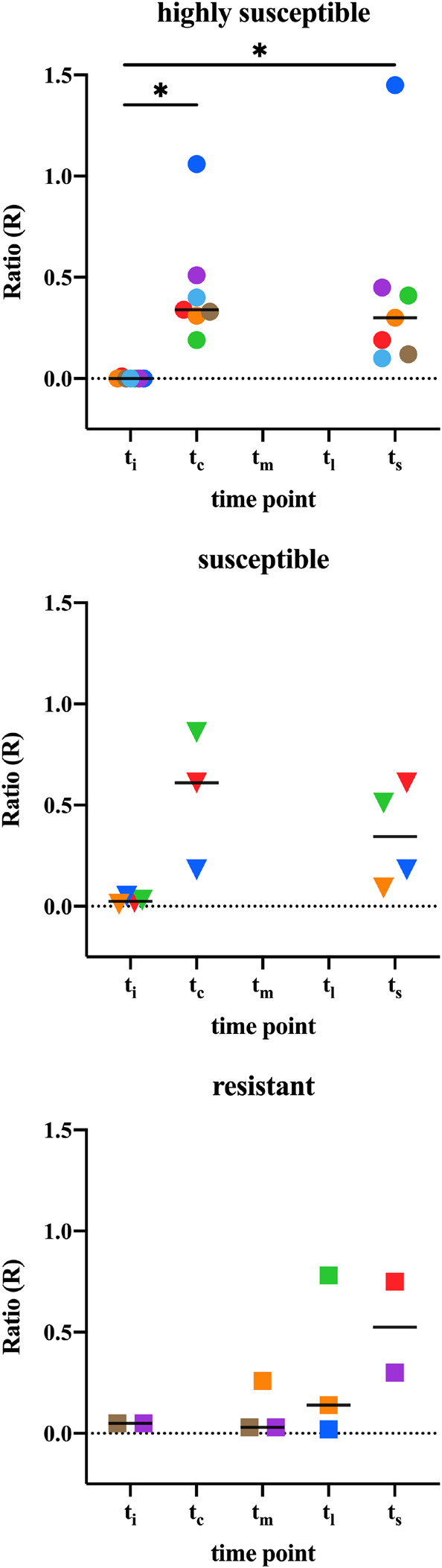
Anti-PGL-I IgM levels by UCP-LFA in M. leprae infected armadillos. Anti-PGL-I IgM levels were measured by UCP-LFA in serum from *M. leprae* [1 × 10^9^ bacilli; NHDP 63 (*n* = 9), NHDP 98 (*n* = 4), or Brazil 4923 (*n* = 4)] infected armadillos divided into highly susceptible (*n* = 12; dots; upper panel), susceptible (*n* = 4; triangles; middle panel), and resistant (*n* = 6; squares, lower panel); sample derived from each animal is represented by the same symbol. UCP-LFA results are displayed as the Ratio value (R) between Test (T) and Flow-Control (FC) signal (*y-*axis). The cut-off value for discrimination between infected vs. uninfected animals is *R* > 0.07. The median values of each group are indicated by horizontal lines. Differences between pre-infection (t_i_) vs. post-infection (t_c_/t_m_/t_l_/t_s_) were determined by Wilcoxon matched-pairs test. *P*-values: ^∗^*p* < 0.05. t_i_, time of inoculation; t_c_, time of seroconversion (for highly susceptible and susceptible animals); t_m_, mid-stage disease; t_l_, late-stage disease; t_s_, time at sacrifice (<12 months for highly susceptible; 12–24 months for susceptible; >36 months for resistant).

To assess whether anti-PGL-I specific antibodies could be detected in infected armadillos using UCP-LFA for detection of anti-PGL-I human IgM (van Hooij et al., 2017, 2020), serum samples (*n* = 41) from *M. leprae* infected armadillos (*n* = 17) were analyzed at multiple timepoints post-infection. As previously observed in humans (van Hooij et al., 2016, 2017), results of the UCP-LFA (Ratio values) correlated well (*R*^2^ = 0.887, *p* < 0.0001) with ELISA data (OD_450__–__background_) (Figure 3). As in the ELISA, anti-PGL-I IgM levels were not detected before *M. leprae* infection. After infection, the anti-PGL-I antibodies were detectable by UCP-LFA and significantly increased in time in the highly susceptible- and susceptible- armadillos (t_c_/t_s_), whereas no relevant difference in Ratio values were observed by UCP-LFA in resistant armadillos before the experimental endpoint (t_i_ vs. t_m_/t_l_) (Figure 4).

### Monitoring Active *M. leprae* Infection in Armadillos

**FIGURE 5:**
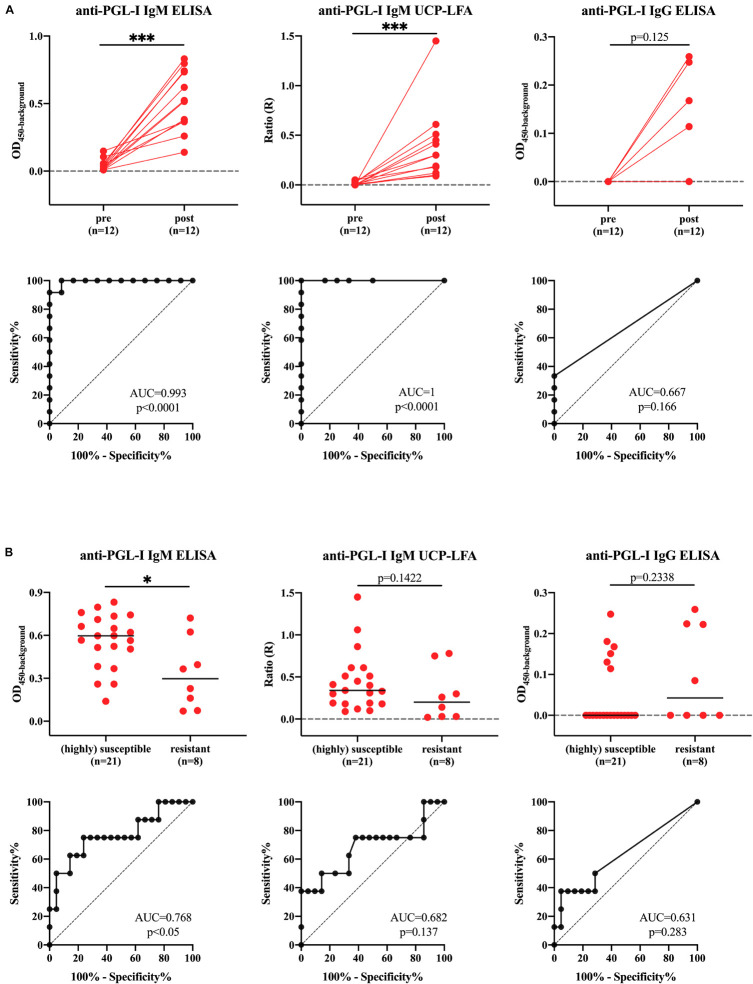
Differences in anti-PGL-I antibody levels between uninfected- and M. leprae infected highly susceptible, susceptible and resistant armadillos. Anti-PGL-I antibody levels were measured by UCP-LFA (IgM) and ELISA (IgM/IgG) in serum from armadillos infected with 1 × 10^9^ *M. leprae* bacilli of the following strains: NHDP 63 (*n* = 9), NHDP 98 (*n* = 4), or Brazil 4923 (*n* = 4). Samples were analyzed pre-infection (t_i_) for all animals (*n* = 21) and post-infection (t_c_/t_m_/t_l_/t_s_) for highly susceptible (*n* = 7), susceptible (*n* = 4) and resistant (*n* = 6) armadillos UCP-LFA results are displayed as the Ratio value (R; *y*-axis) between Test (T) and Flow-Control (FC) signal; ELISA results are displayed as optical density at 450 nm corrected by the background for each sample (OD_450__–__background_). **(A)** For animals for which both samples were available, significant differences between levels in pre (t_i_)- vs. post (t_s_)-infection sera were determined by Wilcoxon matched-paired tests; **(B)** Antibody levels in all sera after inoculation were determined by Mann-Whitney *U*-tests; *P*-values: ^∗^*p* < 0.05, ^∗∗∗^*p* < 0.001. t_i_: time of inoculation; t_c_: time of seroconversion (for highly susceptible and susceptible animals); t_m_, mid-stage disease; t_l_, late-stage disease; t_s_, time at sacrifice.

To assess the potential of the UCP-LFA and ELISA to detect intraindividual levels in anti-PGL-I antibodies, we selected 12 out 17 animals with both pre (t_i_)- and post (t_s_)-infection sampling time points from the above mentioned armadillos for further analysis [highly susceptible (*n* = 7)-, susceptible (*n* = 4)-, resistant-armadillos (*n* = 1)] (Supplementary Table 2). The anti-PGL-I antibody levels were significantly increased post-infection and good discriminatory performance was obtained for anti-PGL-I IgM ELISA and -UCP-LFA with ROC-AUC values of 0.993 (*p* < 0.0001) and 1.000 (*p* < 0.0001) (Figure 5A and Table 1A).

**TABLE 1A*:** Assay performance to discriminate pre- and post-infection (*n* = 12)*.

Assay	Cut-off	Sensitivity (%)	95% CI (%)	Specificity (%)	95% CI (%)	*P*-value
Anti-PGL-I IgM ELISA	OD_450__–__background_ > 0.1228	100.00	75.75–100.00	91.67	64.61–99.57	<0.0001
Anti-PGL-I IgM UCP-LFA	R > 0.07	100.00	75.75–100.00	100.00	75.75–100.00	<0.0001
Anti-PGL-I IgG ELISA	na					

In order to determine whether anti-PGL-I antibody levels can distinguish differential susceptibility to dissemination, we further analyzed all 17 armadillos, including (highly) susceptible (*n* = 11) and resistant (*n* = 6) armadillo samples at all available time points (t_c_/t_m_/t_l_/t_s_) after inoculation (21 samples of (highly) susceptible armadillos, 8 samples of resistant armadillos, respectively) (Supplementary Table 2). The anti-PGL-I IgM antibody levels detected by ELISA and UCP-LFA were more increased after infection in (highly) susceptible animals compared to resistant animals (*p* < 0.05, *p* = 0.1422, respectively). Based on the anti-PGL-I levels, the (highly) susceptible- could be discriminated from the resistant armadillos (AUC = 0.7679, *p* < 0.05) (Figure 5B and Table 1B).

**TABLE 1B:** Assay performance to discriminate (highly) susceptible and resistant armadillos (*n* = 17)*.

Assay	Cut-off	Sensitivity (%)	95% CI (%)	Specificity (%)	95% CI (%)	*P*-value
Anti-PGL-I IgM ELISA	OD_450__–__background_ > 0.45	75	40.93–95.56	76.19	54.91–89.37	<0.05
Anti-PGL-I IgM UCP-LFA	na					
Anti-PGL-I IgG ELISA	na					

*The ability to distinguish different infection states was evaluated by ROC curve analysis, including Area Under the Curve (AUC) measurements (ROC-AUC). **(A)** Assay performance to discriminate pre- and post-infection was performed for samples derived from animals of which both t_i_ and t_s_ were available (n = 12).*

***(B)** Assay performance to discriminate (highly) susceptible and resistant armadillos (n = 17). t_i_, time of inoculation; t_c_, time of seroconversion (for highly susceptible and susceptible animals); t_m_, mid-stage disease; t_l_, late-stage disease; t_s_, time at sacrifice; CI, confidence interval; na, not applicable.*

*The study flowchart of all samples is described in Figure 1 and more detailed description on in the legend of Figure 5.

Since anti-PGL-I IgG levels were not detectable in most samples (20 out of 29) from infected armadillos, discrimination based on anti-PGL-I IgG levels was not possible (Table 1 and Figure 5).

### Anti-PGL-I Antibodies in Vaccinated and Rifampin Treated Armadillos

**FIGURE 6:**
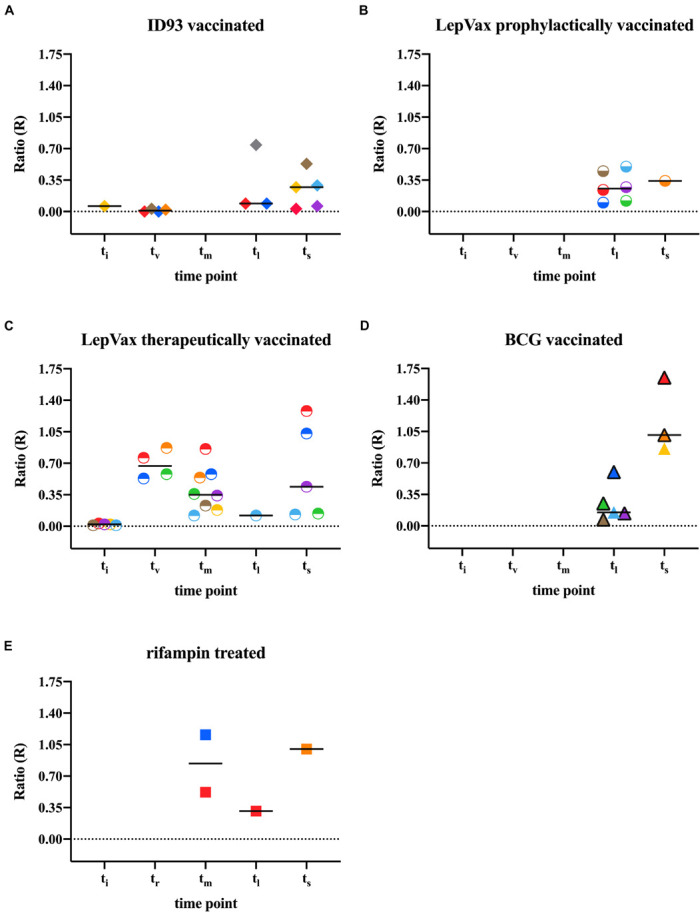
Anti-PGL-I IgM levels by UCP-LFA in M. leprae infected treated/vaccinated armadillos. Anti-PGL-I IgM levels were measured by UCP-LFA in serum from *M. leprae* [1 × 10^9^ bacilli; NHDP 63 (*n* = 24), NHDP 98 (*n* = 3), or Brazil 4923 (*n* = 7)], and one animal was infected in the wild) infected armadillos (*n* = 35). 16 armadillos were prophylactically vaccinated with ID93 (*n* = 9; **A**) or LepVax (*n* = 7, **B**) 1 month post-infection; eight armadillos were therapeutically vaccinated with LepVax **(C)** 10–32 months post-infection; Eight armadillos were vaccinated with BCG **(D)** 1 month pre- (*n* = 6, black frame) or post- (*n* = 2, colored triangle) infection; Rifampin was provided for 3 months to armadillos (*n* = 3) 8 or 10 months post-infection **(E)**. Sample derived from each animal is represented by the same symbol. UCP-LFA results are displayed as the Ratio value (R; *y-axis*) between Test (T) and Flow-Control (FC) signal. The median values of each group are indicated by horizontal lines. Differences in antibody levels between pre-infection (t_i_) vs. post-infection (t_v_/t_r_/t_m_/t_l_/t_s_) were determined by Mann-Whitney *U*-tests. t_i_, time of inoculation; t_v_, time of vaccination; t_r_, time of rifampin treatment; t_m_, mid-stage disease; t_l_, late-stage disease; t_s_, time at sacrifice.

**FIGURE 7:**
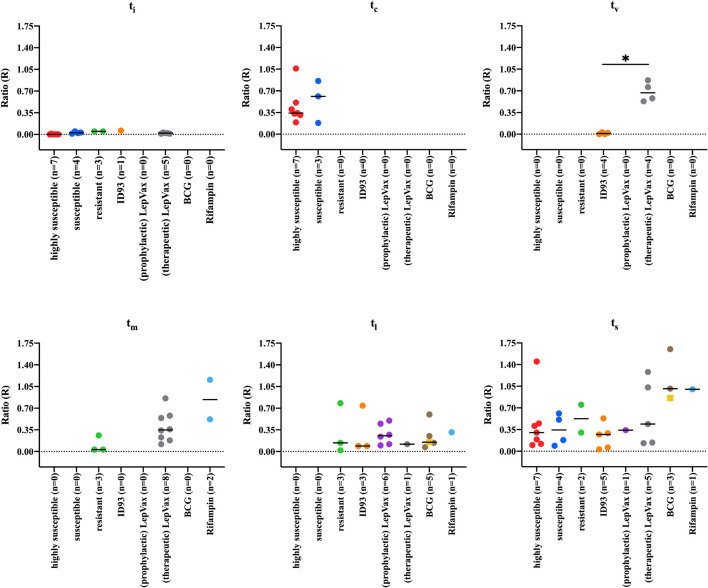
Anti-PGL-I IgM levels by UCP-LFA in M. leprae infected armadillos after interventions. Anti-PGL-I IgM levels were measured by UCP-LFA in serum from *M. leprae* [1 × 10^9^ bacilli; NHDP 63 (*n* = 33), NHDP 98 (*n* = 7), or Brazil 4923 (*n* = 11)], and one animal was infected in the wild) infected armadillos (*n* = 17) divided into highly susceptible (*n* = 7, red dots), susceptible (*n* = 4, blue dots), and resistant (*n* = 6, green dots). 24 armadillos were prophylactically vaccinated with ID93 (*n* = 9, orange) or LepVax (*n* = 7, purple dots) 1 month post-infection; eight armadillos were therapeutically vaccinated LepVax (gray dots) 10–32 months post-infection; Eight armadillos were vaccinated with BCG 1 month pre- (*n* = 6, brown dots) or post- (*n* = 2, yellow squares) infection. Rifampin was provided for 3 months to armadillos (*n* = 3, teal dots) 8 or 10 months post-infection. UCP-LFA results are displayed as the Ratio value (R) between Test (T) and Flow-Control (FC) signal (*y-axis*). The median values of each group are indicated by horizontal lines. Differences between groups were determined by Kruskal-Wallis test with Dunn’s correction for multiple testing. *P*-values: ^∗^*p* < 0.05. t_i_, time of inoculation; t_c_, time of seroconversion (for highly susceptible and susceptible animals); t_v_, time of vaccination; t_m_, mid-stage disease; t_l_, late-stage disease; t_s_, time at sacrifice.

To determine whether the levels of anti-PGL-I antibody measured by UCP-LFA could detect changes reflecting efficient vaccination or drug treatment, we analyzed samples from 24 armadillos that were prophylactically vaccinated with ID93, LepVax, or BCG 1 month pre- (*n* = 2 for BCG) or post- (*n* = 9, 7, 6 for ID93, LepVax, BCG, respectively) experimental *M. leprae* infection. We also added a therapeutically vaccinated group, which included eight armadillos that were vaccinated with LepVax 10–32 months post-infection. In addition, three armadillos were included that were treated for 3 months with rifampin 8 or 10 months post-infection (Supplementary Table 2). Armadillos vaccinated with ID93 or LepVax had less bacilli in the liver and spleen compared to highly susceptible- and susceptible animals, but still a higher bacillary load than resistant animals at the endpoint (Supplementary Figure 1). Anti-PGL-I antibody levels in armadillos prophylactically vaccinated with LepVax (*n* = 1) or ID93 (*n* = 5), were similar to those in untreated, infected animals at the time of sacrifice. However, anti-PGL-I antibody levels in the two vaccinated groups (median t_s_: 1,137 and 1,342 days, respectively) were comparable to those in resistant armadillos (median t_s_: 1,215 days) as dissemination took considerably longer than in (highly) susceptible armadillos (t_s_ median = 292 days). This indicates that both ID93 and LepVax prophylactic vaccination can delay the increase of anti-PGL-I antibody levels and thus bacterial load/disease progression as observed in the resistant animals (Figures 6A,B, 7 and Supplementary Table 2). At the timepoint of sacrifice, anti-PGL-I antibody levels of armadillos that were therapeutically vaccinated with LepVax (*n* = 5), were similar to those of the prophylactically vaccinated animals, which was also reflected by the prolonged time to severe disease (t_s_ median = 981 days) (Figures 6C, 7 and Supplementary Table 2).

After BCG vaccination, armadillos had slightly lower bacillary levels than highly susceptible animals, but higher than ID93 and LepVax vaccinated animals (Supplementary Figure 1). The anti-PGL-I antibody levels detected after BCG (*n* = 5) remained at similarly low levels as after ID93 (*n* = 3) vaccination at t_l_. Furthermore, the antibody levels observed at t_s_ after BCG vaccination (*n* = 3), were higher than after ID93 or LepVax vaccination (Figures 6D, 7), and a shorter time to sacrifice due to *M. leprae* dissemination was observed (t_s_ median = 469 days, Supplementary Table 2). This may indicate that defined subunit vaccines such as LepVax and ID93 are more effective than BCG in reducing *M. leprae* load.

After rifampin treatment of *M. leprae* infected armadillos (*n* = 2), anti-PGL-I antibody levels were increased compared to those in resistant armadillos (*n* = 3) at t_m_, and declined at t_l_ to levels similar to what was observed for resistant armadillos (*n* = 1), reflecting the therapeutic effect of rifampin on *M. leprae* infection. At time of sacrifice (t_s_), rifampin treated armadillos (*n* = 1) showed higher anti-PGL-I antibody levels than untreated susceptible armadillos, although the bacillary load was comparable (Figures 6E, 7 and Supplementary Figure 1). Rifampin treatment for 3 months likely did not completely sterilize *M. leprae* infected armadillos, allowing residual bacteria to continue replicating, but rifampicin treatment still prolonged the time to full dissemination of infected animals (t_s_ median = 674 days). Thus, anti-PGL-I IgM UCP-LFA can monitor the efficacy of vaccination and rifampin treatment in *M. leprae* infected armadillo.

## Discussion

IgM antibodies against *M. leprae*-PGL-I are specific for infection with mycobacteria causing leprosy (Hunter and Brennan, 1981; van Dijk et al., 2021) and anti-PGL-I IgM levels have been amply shown to be quantitively associated with bacterial load in leprosy patients (Zenha et al., 2009; van Hooij et al., 2017; Tió-Coma et al., 2020). However, the presence of these antibodies does not reveal sufficient information about the timing of infection in humans. The wide spectrum of immunology and histopathology in armadillos is similar to human leprosy, which renders this animal model very useful for research on diagnostic markers for active *M. leprae* infection and leprosy disease (Job, 1991; Sharma et al., 2013; Truman et al., 2014). Studies on experimentally infected armadilllos have previously shown that lower ratios between IgM/IgG anti-PGL-I antibodies indicate lower susceptibility for *M. leprae* dissemination in armadillos (Vadiee et al., 1988). In the current study the serological monitoring of anti-PGL-I antibodies in serum of experimentally *M. leprae* infected armadillos with or without vaccination allowed the examination of the kinetics of IgM and IgG anti-PGL-I antibodies early after inoculation.

In humans, detection of *M. leprae* infection before clinical symptoms have occurred is vital to reduce transmission but also to reduce leprosy-associated disabilities. Scarcity of laboratory infrastructure in, often resource limited, leprosy endemic areas, demand low complexity tests that cannot only detect *M. leprae* infection at early stages, but also identify which levels are associated with active infection, and determine leprosy phenotypes at point-of-care and genuine near-patient level. Better insight into the kinetics of antibody formation, particularly directly after *M. leprae* infection, is required for full evaluation of newly developed diagnostic tools (rapid tests) and can be provided by the armadillo leprosy model.

Previously we have shown that a rapid LFA utilizing the luminescent UCP label for detection and quantitation of human anti-PGL-I IgM (van Hooij et al., 2016, 2017, 2018, 2019, 2021), also allows detection of antibodies against PGL-I in *M. leprae* infected red squirrels (Truman et al., 1986; Gormus et al., 1995; da Silva et al., 2018; Schilling et al., 2019, 2021; da Silva Ferreira et al., 2020). The protected status of the red squirrels in the United Kingdom, however, precluded any experiments on kinetics directly after *M. leprae* infection.

In this study, we have successfully examined the use of the anti-PGL-I UCP-LFA in a more assessable animal model by testing sera of nine-banded armadillos and measured longitudinal changes in anti-PGL-I antibody levels in experimentally infected armadillos with different susceptibility to *M. leprae*.

Our data show that the anti-PGL-I UCP-LFA can be used to detect *M. leprae* infection and even monitor the efficacy of vaccination and rifampin-treatment in susceptible armadillos. Thus, as demonstrated in the armadillo model for leprosy, the UCP-LFA provides a low-complexity tool for detection of infection and is a promising monitoring tool to evaluate the efficacy of vaccines and drugs.

Anti-PGL-I UCP-LFA data were only weakly correlated with other blood indicators that were used for bacterial load and severity evaluation of leprosy in the armadillos, such as serum lactate dehydrogenase (LDH), aspartate aminotransferase (AST), and alanine aminotransferase (ALT) (Rojas-Espinosa et al., 1985). This emphasizes the improved diagnostic value of anti-PGL-I IgM UCP-LFA as a biomarker for active *M. leprae* infection, especially since anti-PGL-I IgM could be detected in all infected animals.

In humans anti-PGL-I IgM has been reported on more often in leprosy research than anti-PGL-I IgG or -IgA (Pierneef et al., 2021). Monitoring the dynamic differences of anti-PGL-I IgM and IgG antibodies in sera from experimentally *M. leprae* infected armadillo provides insight into the formation and kinetics of these antibodies in humans at the early stage of infection. The current study showed that anti-PGL-I IgM as well as IgG antibodies increased after *M. leprae* infection. Both antibody types were associated with susceptibility to infection, which was most apparent for the IgM isotype which increased rapidly in highly susceptible- and susceptible animals. The UCP-LFA could detect early phases of *M. leprae* infection (after 140 days) with 100% sensitivity and specificity comparing pre- to post-infection. However, since only armadillos showing a negative lepromin response (indicating susceptibility to *M. leprae*) were experimentally infected with *M. leprae* in this study, we were not able to assess whether the UCP-LFA can be used to discriminate susceptible from resistant armadillos as is the case for MB and PB leprosy in humans (van Hooij et al., 2017).

Anti-PGL-I IgG was only detected in a limited number of samples and also more frequently in resistant animals. This may indicate that the anti-PGL-I IgM and IgG play different roles in anti-*M. leprae* humoral immunity and vary in dynamic changes during *M. leprae* infection. The IgM response, however, was long-lasting in all animals in which IgM antibodies were observed after infection and did not wane over time (maximal 1,325 days in resistant animals). This is similar to our observation in squirrels, as IgM levels specifically increased in red squirrels developing leprosy symptoms (Schilling et al., 2021). In both animal models anti-PGL-I specific IgM was still present at higher levels years after infection, indicating that these antibodies not only indicate recent *M. leprae* infection. These data corroborate the finding that in human leprosy the presence of IgM antibodies does not only indicate recent infection.

Determining the ratio of the two types of antibodies could advance leprosy diagnosis in humans, particularly for active infection and disease classification.

Since armadillos infected with *M. leprae* are a potential environmental reservoir for the zoonotic transmission of leprosy (Truman et al., 2011; Sharma et al., 2015; da Silva et al., 2018; da Silva Ferreira et al., 2020), detecting *M. leprae* infection in armadillos is essential to eliminate the spread of leprosy. The UCP-LFA is a minimally invasive, rapid, low-cost, and user-friendly test. This study has demonstrated that UCP-LFA can effectively detect anti-PGL-I antibodies in armadillos which correlated well with ELISA for IgM. The anti-PGL-I IgM by UCP-LFA could be applied as a low complexity screening tool to detect environmental reservoirs of *M. leprae*. Since in this study only armadillos showing a lepromatous leprosy (LL) type response to lepromin were included, representing the majority of the *M. leprae* infected armadillos (Job et al., 1983; Job and Truman, 2000), follow-up studies should also include armadillos with tuberculoid leprosy (TT) responses to lepromin.

In summary, this study shows that the UCP-LFA allows quantitative measurements of anti-PGL-I levels in serum of nine-banded armadillos directly after experimental *M. leprae* infection, allowing discrimination between susceptible and resistant animals. In view of the spectral pathology of armadillos, this animal model provides useful insight into antibody kinetics in early infection and spectral forms of human leprosy. The armadillo leprosy model indicates that the UCP-LFA detecting anti-PGL-I IgM can be used for detection of infection in humans at early stages as well as monitoring changes in bacillary load. Thus, this UCP-LFA provides a rapid and low-cost tool to screen for infection and evaluate the effect of prophylactic and therapeutic treatments (Richardus et al., 2019; Richardus and Geluk, 2020).

## Data Availability Statement

The original contributions presented in the study are included in the article/Supplementary Material, further inquiries can be directed to the corresponding author/s.

## Ethics Statement

The animal study was reviewed and approved by the NHDP Animal Care and Use Committee.

## Author Contributions

AG: conceptualization. AG, AH, MP, and ZZ: data curation. RS and RW: animal experiments. DJ and LP: production and QC UCP-LFA. AG, AH, LP, and ZZ: formal analysis. AG and ZZ: writing—original draft. AG, AH, LA, MP, PC, RT, and ZZ: writing—review and editing. All authors agreed with manuscript results and conclusion.

## Conflict of Interest

The authors declare that the research was conducted in the absence of any commercial or financial relationships that could be construed as a potential conflict of interest. The reviewer WL declared a past collaboration with one of the author MP to the handling editor.

## Publisher’s Note

All claims expressed in this article are solely those of the authors and do not necessarily represent those of their affiliated organizations, or those of the publisher, the editors and the reviewers. Any product that may be evaluated in this article, or claim that may be made by its manufacturer, is not guaranteed or endorsed by the publisher.
