# Supplementary material for: Detection and Monitoring of Mycobacterium leprae Infection in Nine Banded Armadillos (Dasypus novemcinctus) Using a Quantitative Rapid Test
Source: Front Microbiol. 2021 Oct 28;12:763289. doi: 10.3389/fmicb.2021.763289 (PMC8581735; doi:10.3389/fmicb.2021.763289)
Supplement: Supplementary file 1 [file Data_Sheet_1.docx]

Supplementary Material

Detection and Monitoring of *Mycobacterium leprae* Infection in Nine Banded Armadillos (*Dasypus novemcinctus*) Using a Quantitative Rapid Test

**Zijie Zhou^a^, Maria Pena^b^, Anouk van Hooij^a^, Louise Pierneef^a^, Danielle de Jong^c^, Roenna Stevenson^b^, Rachel Walley^b^, Paul L.A.M. Corstjens^c^, Richard Truman^d^, Linda Adams^b^, and Annemieke Geluk^a,*^**

**Table S1. *Characteristics of the armadillos in the study cohort***

| **Group description** | **n** | **vaccination/ treatment** | **reference** |
| --- | --- | --- | --- |
| highly susceptible | 7 | - | (Pena et al., 2016) |
| susceptible | 4 | - | (Pena et al., 2016) |
| resistant | 6 | - | (Pena et al., 2016) |
| ID93 vaccinated | 9 | ID93 one month post-infection | (Duthie et al., 2018) |
| Lepvax vaccinated | 15 | LepVax one month post-infection (n = 7)  LepVax 10-32 months post-infection (n = 8) | (Duthie et al., 2018) and unpublished data |
| BCG vaccinated | 8 | BCG one month pre-infection (n = 6)  BCG one month post-infection (n = 2) | unpublished data |
| rifampin treated | 4 | rifampin eight months post-infection (n = 2)  rifampin ten months post-infection (n = 2) | unpublished data |

Armadillos (n = 53) were infected *M. leprae* (1x10^9^ bacilli, NHDP 63 (n = 33), NHDP 98 (n = 7), or Brazil 4923 (n = 11), and one animal was infected in the wild) by saphenous vein injection. Untreated/ unvaccinated armadillos (n = 17) were divided into three group based on the time elapsed after infection until dissemination occurred: high susceptible (within 12 months), susceptible (within 24 months), and resistant (more than 36 months); 32 armadillos were vaccinated with ID93, LepVax or BCG 1-32 month pre- or post-infection; three armadillos were treated for three months with rifampin post-infection. “-” indicates unvaccinated or untreated.

**Supplementary Table S2: *Characteristics of armadillo study cohort***

| **Animal number** | **Experiment group** | ***M. leprae* strain** | **Vaccinated/treated time** | **Sampling time points (days)** | | | | | | |
| --- | --- | --- | --- | --- | --- | --- | --- | --- | --- | --- |
|  |  |  |  | **t_i_** | **t_v_** | **t_r_** | **t_c_** | **t_m_** | **t_l_** | **t_s_** |
| 1 | highly susceptible | NHDP98 | na | 0 |  |  | 140 |  |  | 192 |
| 2 | highly susceptible | NHDP98 | na | 0 |  |  | 168 |  |  | 206 |
| 3 | highly susceptible | NHDP98 | na | 0 |  |  | 152 |  |  | 157 |
| 4 | highly susceptible | NHDP98 | na | 0 |  |  | 140 |  |  | 222 |
| 5 | highly susceptible | Brazil4923 | na | 0 |  |  | 278 |  |  | 292 |
| 6 | highly susceptible | Brazil4923 | na | 0 |  |  | 244 |  |  | 318 |
| 7 | highly susceptible | NHDP63 | na | 0 |  |  | 208 |  |  | 244 |
| 8 | susceptible | NHDP63 | na | 0 |  |  | 294 |  |  | 519 |
| 9 | susceptible | NHDP63 | na | 0 |  |  | 294 |  |  | 437 |
| 10 | susceptible | Brazil4923 | na | 0 |  |  | 361 |  |  | 361 |
| 11 | susceptible | Brazil4923 | na | 0 |  |  |  |  |  | 385 |
| 12 | resistant | NHDP63 | na |  |  |  |  |  |  | 1105 |
| 13 | resistant | NHDP63 | na |  |  |  |  |  | 1310 |  |
| 14 | resistant | NHDP63 | na |  |  |  |  |  | 1310 |  |
| 15 | resistant | NHDP63 | na |  |  |  |  | 268 | 629 |  |
| 16 | resistant | NHDP63 | na | 0 |  |  |  | 400 |  |  |
| 17 | resistant | NHDP63 | na | 0 |  |  |  | 400 |  | 1325 |
| 18 | ID93 vaccinated | NHDP63 | 1 month post-infection |  | 30 |  |  |  | 498 |  |
| 19 | ID93 vaccinated | NHDP63 | 1 month post-infection |  | 30 |  |  |  | 498 |  |
| 20 | ID93 vaccinated | NHDP63 | 1 month post-infection |  | 30 |  |  |  |  |  |
| 21 | ID93 vaccinated | NHDP63 | 1 month post-infection |  | 30 |  |  |  |  | 1342 |
| 22 | ID93 vaccinated | NHDP63 | 1 month post-infection |  |  |  |  |  |  | 1342 |
| 23 | ID93 vaccinated | NHDP63 | 1 month post-infection |  |  |  |  |  |  | 1342 |
| 24 | ID93 vaccinated | NHDP63 | 1 month post-infection |  |  |  |  |  | 164 |  |
| 25 | ID93 vaccinated | NHDP63 | 1 month post-infection | 0 |  |  |  |  |  | 1173 |
| 26 | ID93 vaccinated | NHDP63 | 1 month post-infection |  |  |  |  |  |  | 1169 |
| 27 | LepVax vaccinated | NHDP63 | 1 month post-infection |  |  |  |  |  | 476 |  |
| 28 | LepVax vaccinated | NHDP63 | 1 month post-infection |  |  |  |  |  | 1155 |  |
| 29 | LepVax vaccinated | NHDP63 | 1 month post-infection |  |  |  |  |  | 446 |  |
| 30 | LepVax vaccinated | NHDP63 | 1 month post-infection |  |  |  |  |  |  | 1137 |
| 31 | LepVax vaccinated | NHDP63 | 1 month post-infection |  |  |  |  |  | 167 |  |
| 32 | LepVax vaccinated | NHDP63 | 1 month post-infection |  |  |  |  |  | 174 |  |
| 33 | LepVax vaccinated | NHDP63 | 1 month post-infection |  |  |  |  |  | 747 |  |
| 34 | LepVax vaccinated | Brazil4923 | 28 months post-infection | 0 | 855 |  |  | 941 |  | 1072 |
| 35 | LepVax vaccinated | Brazil4923 | 28 months post-infection |  | 855 |  |  | 941 |  | 981 |
| 36 | LepVax vaccinated | NHDP98 | 32 months post-infection |  | 966 |  |  | 1062 |  | 1272 |
| 37 | LepVax vaccinated | na | na (infected in wild) | unknown | t_v_ |  |  | t_v_+86 |  |  |
| 38 | LepVax vaccinated | Brazil4923 | 10 months post-infection | 0 |  |  |  | 371 |  |  |
| 39 | LepVax vaccinated | Brazil4923 | 10 months post-infection | 0 |  |  |  | 371 |  |  |
| 40 | LepVax vaccinated | Brazil4923 | 10 months post-infection | 0 |  |  |  | 371 |  | 594 |
| 41 | LepVax vaccinated | Brazil4923 | 10 months post-infection | 0 |  |  |  | 371 | 594 | 678 |
| 42 | BCG vaccinated | NHDP63 | 1 month pre-infection |  |  |  |  |  |  | 469 |
| 43 | BCG vaccinated | NHDP63 | 1 month pre-infection |  |  |  |  |  | 349 |  |
| 44 | BCG vaccinated | NHDP63 | 1 month pre-infection |  |  |  |  |  | 328 |  |
| 45 | BCG vaccinated | NHDP63 | 1 month pre-infection |  |  |  |  |  |  | 350 |
| 46 | BCG vaccinated | NHDP63 | 1 month pre-infection |  |  |  |  |  | 328 |  |
| 47 | BCG vaccinated | NHDP63 | 1 month pre-infection |  |  |  |  |  | 328 |  |
| 48 | BCG vaccinated | NHDP63 | 1 month post-infection |  |  |  |  |  |  | 1106 |
| 49 | BCG vaccinated | NHDP63 | 1 month post-infection |  |  |  |  |  | 248 |  |
| 50 | rifampin treated | NHDP98 | 8 months post-infection |  |  |  |  | 351 |  |  |
| 51 | rifampin treated | NHDP98 | 8 months post-infection |  |  |  |  | 319 | 351 |  |
| 52 | rifampin treated | Brazil4923 | 10 months post-infection |  |  |  |  |  |  | 674 |

Blood samples (n = 96) of armadillos (n = 52) infected with *M. leprae* (1x10^9^ bacilli, NHDP 63 (n = 33), NHDP 98 (n = 7), or Brazil 4923 (n = 11), and one animal was infected in the wild) collected at several time points. Infected armadillos (n = 17) that were untreated and unvaccinated were divided into 3 groups according to *M. leprae* dissemination and disease progression: highly susceptible (within 12 months; n = 7), susceptible (within 24 months; n = 4), and resistant (after more than 36 months; n = 6); eight armadillos were vaccinated with BCG one month pre- (n = 6) or post- (n = 2) infection; 16 armadillos were prophylactically vaccinated with ID93 (n = 9) or LepVax (n = 7) one month post-infection; eight armadillos were therapeutically vaccinated LepVax (n = 8) 10-32 months post-infection; three armadillos were treated for three months with rifampin 8 or 10 months post-infection. For armadillo #10 (susceptible), t_m_, t_l_ were the same sample; For armadillo #37 (LepVax vaccinated) which was infected in the wild, the inoculation time (t_i_) and interval between t_i_ and t_v_/t_m_ were unknown). t_i_: time of inoculation; t_v_: time of vaccination; t_r_: time of rifampin treatment; t_c_: time of seroconversion (anti-PGL-I IgM ELISA > 0.45 OD_450-background_; for the highly susceptible and susceptible group); t_m_: mid-stage disease; t_l_: late-stage disease; t_s_: time at sacrifice. na: not applicable.**Supplementary Figure S1**


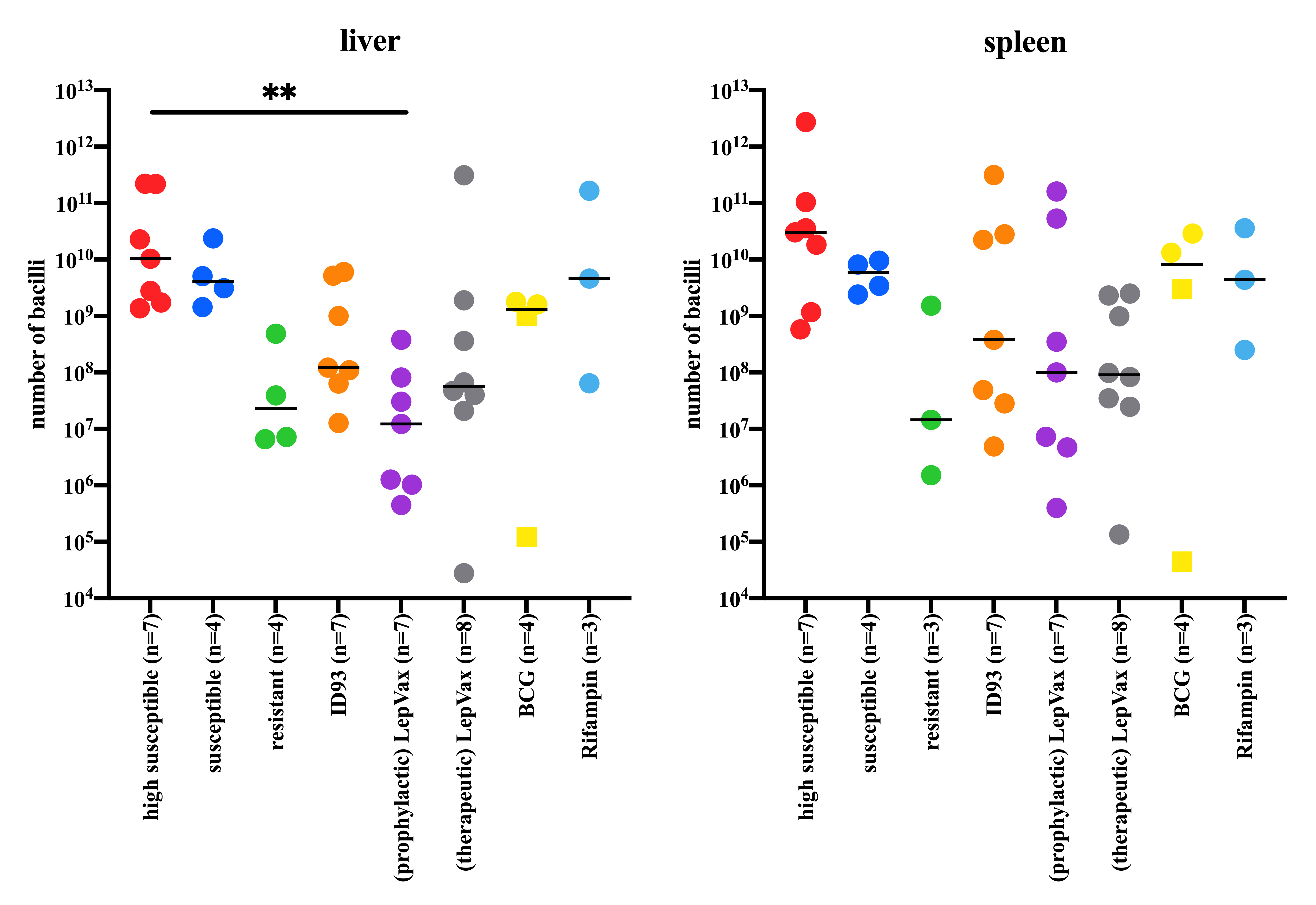


**Figure S1. *Bacillary load in M. leprae infected vaccinated/ treated armadillos***

Bacillary load in the liver (left panel) and spleen (right panel) were measured at the experimental endpoint of *M. leprae* (1x10^9^ bacilli, NHDP 63 (n = 33), NHDP 98 (n = 7), or Brazil 4923 (n = 11), and one animal was infected in the wild) infected armadillos (n = 15) divided into highly susceptible (n = 7, red dots), susceptible (n = 4, blue dots), and resistant (n = 4, green dots). 14 armadillos were prophylactically vaccinated with ID93 (n = 7, orange dots) or LepVax (n = 7, purple dots) one month post-infection; eight armadillos were therapeutically vaccinated LepVax (n = 8, gray dots) 10-32 months post-infection; four armadillos were vaccinated with BCG one month pre- (n = 2, yellow dots) or post- (n = 2, yellow squares) infection. Rifampin was provided for three months to armadillos (n = 3, light blue dots) 8 or 10 months post-infection. The median values of each group are indicated by horizontal lines. Differences between group were determined by Kruskal-Wallis test with Dunn’s correction for multiple testing. P-values: *p < 0.05.

**
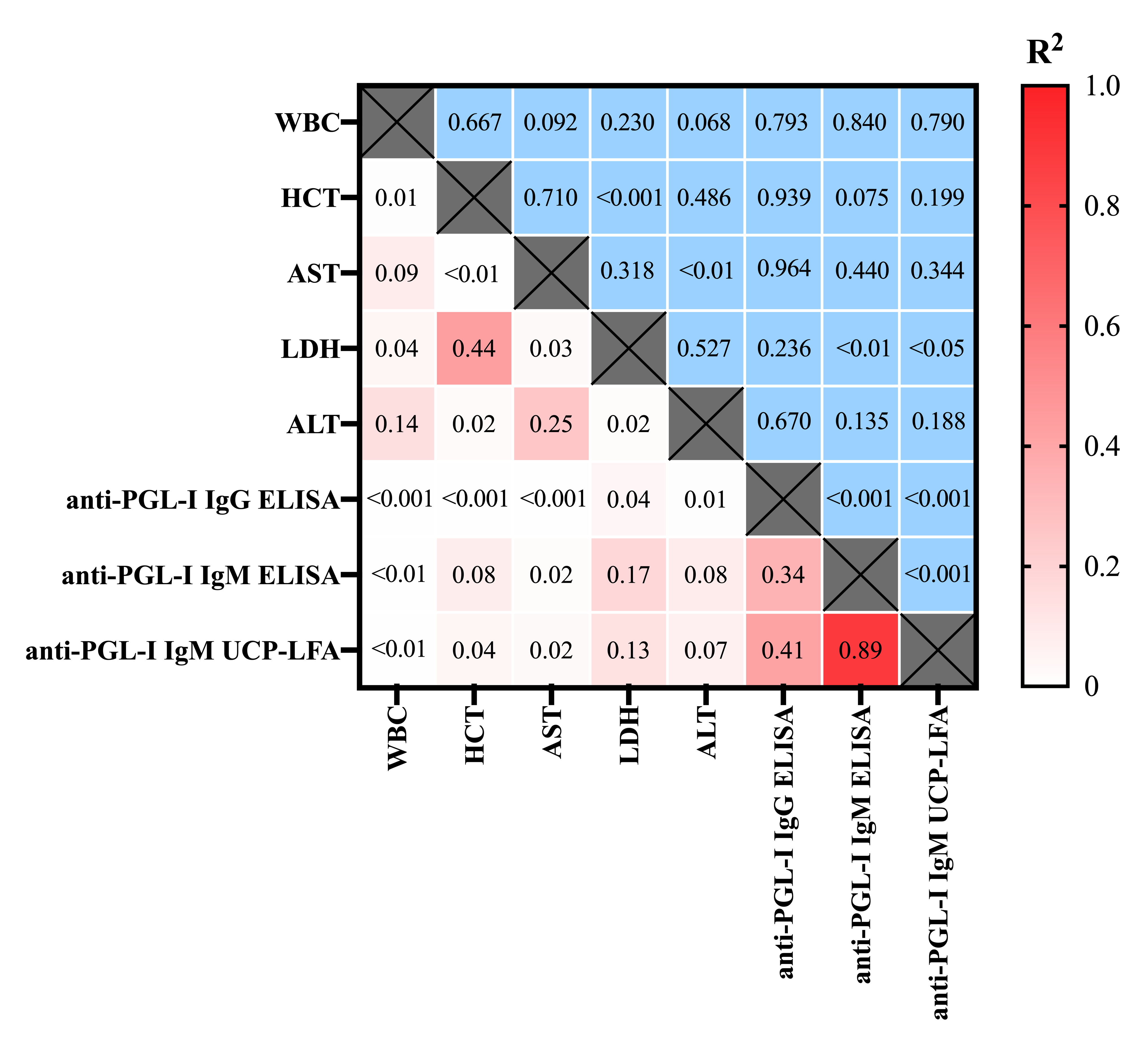
**

**Figure S2: *Correlation between antibody levels and other blood values***

Serum samples (n = 41) of *M. leprae* (1x10^9^ bacilli, NHDP 63 (n = 9), NHDP 98 (n = 4), or Brazil 4923 (n= 4)) infected armadillos (n = 17) were assessed for anti-PGL-I IgM/-IgG by ELISA and UCP-LFA (IgM). At the time of serum collection, blood samples were also assessed for white blood cell counts (WBC; 10^3^/ul;), hematocrit (HCT; %), lactate dehydrogenase (LDH; Units/L), aspartate aminotransferase (AST; Units/L), and alanine aminotransferase (ALT; Units/L). ELISA results are displayed as optical density at 450 nm (OD_450-backgroud_). UCP-LFA results are depicted as Ratio values (R) which indicates the fluorescence intensity measure at the Test line (T) divided by that at the flow control line (FC). R^2^ values, the square of the Spearman correlation coefficients, are depicted in red. The corresponding p-values, indicating the significance level of the observed correlation between the different tests, are shown in blue.

**References:**

Duthie, M.S., Pena, M.T., Ebenezer, G.J., Gillis, T.P., Sharma, R., Cunningham, K., et al. (2018). LepVax, a defined subunit vaccine that provides effective pre-exposure and post-exposure prophylaxis of *M. leprae* infection. *NPJ Vaccines* 3**,** 12. doi: 10.1038/s41541-018-0050-z.

Pena, M.T., Sharma, R., and Truman, R.W. (2016). *The armadillo model for leprosy* [Online]. Available: <https://www.internationaltextbookofleprosy.org/chapter/armadillos> [Accessed 18 September 2016].
